# Interactions Between the Gravitostat and the Fibroblast Growth Factor System for the Regulation of Body Weight

**DOI:** 10.1210/en.2018-01002

**Published:** 2019-03-19

**Authors:** Vilborg Palsdottir, Sara H Windahl, Daniel A Hägg, Hanna Keantar, Jakob Bellman, Andrew Buchanan, Tristan J Vaughan, Daniel Lindén, John-Olov Jansson, Claes Ohlsson

**Affiliations:** 1 Division of Endocrinology, Department of Neuroscience and Physiology, Sahlgrenska Academy, University of Gothenburg, Gothenburg, Sweden; 2 Centre for Bone and Arthritis Research, Department of Medicine, Sahlgrenska Academy, University of Gothenburg, Gothenburg, Sweden; 3 Division of Pathology, Department of Laboratory Medicine, Karolinska Institutet, Huddinge, Sweden; 4 Antibody Discovery and Protein Engineering, MedImmune Ltd., Cambridge, United Kingdom; 5 Cardiovascular and Metabolic Diseases, Innovative Medicines and Early Development Biotech Unit, AstraZeneca, Gothenburg, Sweden

## Abstract

Both fibroblast growth factors (FGFs), by binding to FGF receptors (FGFRs), and activation of the gravitostat, by artificial loading, decrease the body weight (BW). Previous studies demonstrate that both the FGF system and loading have the capacity to regulate BW independently of leptin. The aim of the current study was to determine the possible interactions between the effect of increased loading and the FGF system for the regulation of BW. We observed that the BW-reducing effect of increased loading was abolished in mice treated with a monoclonal antibody directed against FGFR1c, suggesting interactions between the two systems. As serum levels of endocrine FGF21 and hepatic FGF21 mRNA were increased in the loaded mice compared with the control mice, we first evaluated the loading response in FGF21 over expressing mice with constant high FGF21 levels. Leptin treatment, but not increased loading, decreased the BW in the FGF21-overexpressing mice, demonstrating that specifically the loading effect is attenuated in the presence of high activity in the FGF system. However, as FGF21 knockout mice displayed a normal loading response on BW, FGF21 is neither mediating nor essential for the loading response. In conclusion, the BW-reducing effect of increased loading but not of leptin treatment is blocked by high activity in the FGF system. We propose that both the gravitostat and the FGF system regulate BW independently of leptin and that pharmacologically enhanced activity in the FGF system reduces the sensitivity of the gravitostat.

Obesity is a growing problem worldwide, and it is associated with increased mortality and morbidity (1). Today, there are few effective pharmacological treatment options to decrease obesity or to affect body weight (BW) homeostasis. In 1994, the fat mass regulating hormone leptin was identified (2), but unfortunately, leptin was not successful as a treatment of common obesity in humans (3). Only a few people in the world suffering from leptin deficiency have a good effect from leptin treatment (4). Therefore, leptin has, so far, been of limited clinical use, although the marked obesity caused by leptin deficiency clearly proves the biological importance of leptin (5, 6).

The family of fibroblast growth factors (FGFs) regulates energy metabolism and provides a new approach to the treatment of obesity and other metabolic diseases (7). Some FGFs are released into the circulation and can then act as endocrine hormones. The binding of endocrine FGFs, such as FGF15, FGF21, and FGF23, to their FGF receptors (FGFRs) is promoted via their interactions with coreceptors, such as *β*-Klotho. The modulation of FGFR activity decreases BW independently of leptin (8, 9). Additionally, we have recently published a homeostatic regulation of BW and fat mass, named the gravitostat, which affects the glucose metabolism, and like FGF, it is leptin independent (10, 11). The gravitostat can be activated by an artificial weight-loading treatment, which is effective in the decreasing of BW in mouse and rat. The effect of gravitostat activation seems to be larger in obese animals, contrary to leptin but the mechanism behind is not fully known (10, 11).

Both the family of FGFs (8, 9) and the gravitostat (10) have been shown to regulate metabolism and BW independently of leptin, but there is a lack of knowledge of the interactions between these two systems. By studying the interactions between increased loading and other known systems affecting BW, such as the FGF system and leptin, we may identify novel targets for anti-obesity treatment.

There are several FGFRs, and each receptor may bind several FGFs. The FGFR1c is abundant in the hypothalamus, and one of the FGFs binding to this receptor is FGF21. It has been shown that increased loading during exercise increases hepatic FGF21 production in mice, as well as serum FGF21 levels in both mice and humans (12, 13). The aim of the current study was to determine the possible interactions between the effect of increased loading and the FGF system for the regulation of BW.

## Materials and Methods

### Animals

All animal procedures were approved by the Ethics Committee on Animal Care and Use in Gothenburg, Sweden. C57BL/6 mice were purchased from Taconic (Ejby, Denmark). Transgenic FGF21 mice, FGF21 LoxP mice, and mice expressing Cre recombinase ubiquitously and from an early embryonic stage, under the control of the phosphoglycerate kinase-1 promoter (PGK-Cre), were from The Jackson Laboratory (Bar Harbor, ME). To generate the FGF21 knockout (KO) mice, PGK-Cre mice were crossed with FGF21 LoxP mice. Removal of the PGK-Cre allele and deletion of the FGF21 gene were confirmed by genotyping. Three different primer pairs were used to evaluate which mice had the FGF21 LoxP allele (forward 5′-AAGCATTCCTGGTACCACGG-3′ and reverse 5′-AGCACTAAGGGAGGCAGAGGCAAGTGATT-3′), the deleted FGF21 allele (forward 5′-CCTCCAGATTTAGGAGTGCAG A-3′ and reverse 5′-AGGGAGGCAGAGGCAAGTGATT-3′), and the PGK-Cre allele (forward 5′-AACATGCTTCATCGTCGG-3′ and reverse 5′-TTCGGATCATCAGCTACACC-3′). All genetically modified mice and their littermate controls were on a C57BL/6 background.

### Loading

Mice were fed a high-fat diet (60% fat; D12492; Research Diets, New Brunswick, NJ) during 4 weeks, and then a capsule that weighed 15% of the BW (load) or 3% of the BW (control) was implanted intraperitoneally into the mice under isoflurane anesthesia. The BWs before capsule implantation did not differ between the load and control groups of mice with the same genotype and sex (Table 1). The capsules were made of an inert plastic (Sustarin C; Röchling, Mannheim, Germany) that was shaped as a capsule and filled with tungsten powder (Sigma Aldrich, St. Louis, MO) in the load mice. After tight closure of the capsule by snap-cap construction, it was soaked in chlorhexidine (5 mg/mL; Fresenius Kabi, Bad Homburg, Germany) to disinfect it before implantation. After implantation, the BW was measured several times per week until the end of each experiment.

**Table 1.** BWs Before Surgery for All Experimental Groups Expressed As Means ± SEM

Experimental Group, Mice	Sex	Control Mice BW, g	Load Mice BW, g	*P* Value
FGFR1c ab	Male	38.3 ± 2.0	37.4 ± 1.5	>0.05
IgG ab	Male	39.0 ± 1.4	38.4 ± 1.7	>0.05
Transgenic FGF21	Male	21.7 ± 0.2	21.8 ± 0.3	>0.05
Littermate WT	Male	40.2 ± 0.9	39.9 ± 1.0	>0.05
Transgenic FGF21	Female	18.7 ± 0.3	18.9 ± 0.3	>0.05
Littermate WT	Female	31.0 ± 0.7	31.3 ± 0.7	>0.05
FGF21 KO	Male	35.2 ± 0.9	36.2 ± 1.1	>0.05
Littermate WT	Male	36.8 ± 0.9	37.5 ± 1.0	>0.05
FGF21 KO	Female	32.4 ± 2.1	31.3 ± 1.3	>0.05
Littermate WT	Female	31.9 ± 1.1	31.8 ± 1.2	>0.05

Abbreviation: WT, wild type.

### Leptin treatment

Female transgenic FGF21 mice were injected subcutaneously with leptin [1.5 µg/g BW, twice per day; PeproTech, Rocky Hill, NJ] or saline, on days 21 to 25 after the capsule implantation. The BW was measured every morning during this treatment period.

### FGFR1c antibodies

Antibodies against the FGFR1c (catalog no. R1c monoclonal antibody) (14) or negative control antibodies against IgG (8), obtained from MedImmune Ltd. (Cambridge, UK), were injected subcutaneously (10 µg/g BW) to male C57BL/6 mice with control or load capsules on the same day as the capsule implantation, and the injection was repeated 1 week later.

### Serum analyses

Blood samples were collected from auxiliary blood vessels at the end of each experiment, and the serum was separated and kept in −80°C until analysis. Serum was analyzed by ELISAs for FGF15 (15) (Cloud-Clone Corp., Katy, TX) and FGF21 (16) (R&D Systems, Minneapolis, MN). Furthermore, the serum concentration of six different cytokines (IL-1*β*, IL-6, IL-10, IL-17A, interferon-*γ*, and TNF-*α*) was determined with a bead-based multiplex array kit (17) with Bio-Plex technology, prepared according to the manufacturer’s specifications (Bio-Rad, Hercules, CA).

### Gene expression

Cortical bone from femur and tibia, liver, skeletal muscle (*musculus gastrocnemius*), and inguinal white adipose tissue (WAT) was dissected, snap frozen in liquid nitrogen, and kept in −80°C until analysis. The cortical bones were homogenized with TRIzol reagent (Invitrogen, Carlsbad, CA) before extraction. mRNA, from the cortical bones, liver, and skeletal muscle, was extracted using the RNeasy Mini Kit (Qiagen, Hilden, Germany), and mRNA from the WAT was extracted using the RNeasy Lipid Tissue Mini Kit (Qiagen). The mRNA concentration of the samples was measured by a NanoDrop spectrophotometer (Wilmington, DE), and cDNA was synthesized from 1 µg mRNA with an iScript cDNA synthesis kit (Bio-Rad).

Real-time PCR was performed using the StepOnePlus Real-Time PCR System (Applied Biosystems, Foster City, CA). The liver, WAT, skeletal muscle, and cortical bone samples were analyzed with an assay for FGF21 (Mm00840165_g1). All samples were normalized to 18S (4310893E). The relative mRNA levels were obtained by use of 2-^ΔΔCT^ and calculated with the ΔΔCT equation (18).

### Statistics

Data were analyzed using Student *t* test between control and load groups and between leptin and saline groups. Normality of data was analyzed by the Kolmogorov-Smirnov test, and data were adjusted by log transformation when needed to reach normality. *P* < 0.05 was considered statistically significant. All data are presented as means ± SEM.

## Results

### The effect of increased loading on BW is depleted by interference with FGFR1c

**Figure 1.**
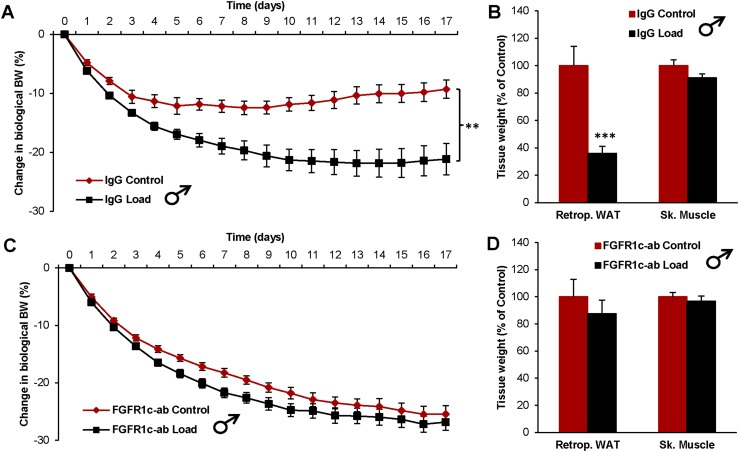
The effect of increased loading on BW is depleted by interference with FGFR1c. Effect of loading on (A) the change in biological BW and (B) tissue weights in IgG-treated mice (control n = 10, and load n = 10). Effect of loading on (C) the change in biological BW and (D) tissue weights in FGFR1c antibody (ab)–treated mice (control n = 10, and load n = 9). Data are expressed as means ± SEM. **P < 0.01; ***P < 0.001. Retrop., retroperitoneal; Sk. Muscle, skeletal muscle (*musculus gastrocnemius*).

To determine if the FGF system interacts with the effect of increased loading, we first investigated the effect of loading on BW in mice treated with an FGFR1c antibody. The IgG-treated control mice responded to loading with a decreased BW and body fat (Fig. 1A and 1B). In contrast, treatment with a monoclonal antibody, directed against FGFR1c, completely blocked these effects of loading on BW and body fat (Fig. 1C and 1D).

### Loading increases FGF21 levels in serum and hepatic FGF21 mRNA

**Figure 2.**
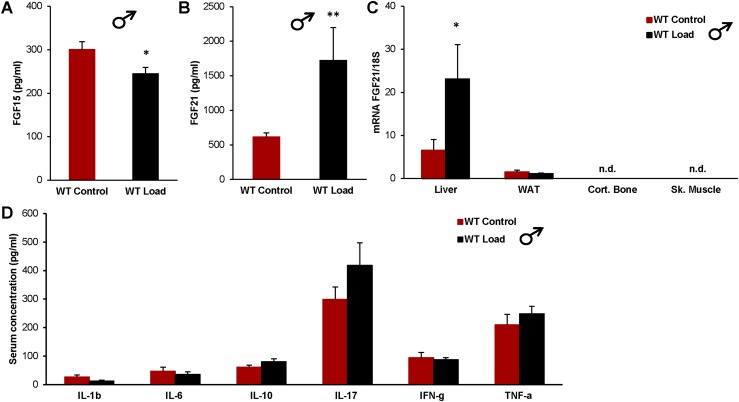
Loading increases FGF21 levels in serum and hepatic FGF21 mRNA. Effects of increased loading in WT male mice on (A) serum levels of FGF15; (B) serum levels of FGF21; (C) mRNA levels of FGF21/18S in liver, WAT, cortical bone (Cort. Bone), and skeletal muscle (Sk. Muscle); and (D) serum levels of cytokines, 6 days after implantation of control or load capsules. Data are expressed as means ± SEM. *P < 0.05; **P < 0.01. n.d., not detectable; WT, wild-type.

We next evaluated if increased loading altered the serum levels of any of the three endocrine FGFs: FGF15, FGF23, and FGF21. The FGF15 levels were slightly decreased in the loaded mice (Fig. 2A), and we have previously shown that there was no difference among the groups in FGF23 levels (10). However, the most pronounced difference was found in the serum levels of FGF21, which were increased threefold in the loaded mice compared with controls (Fig. 2B). We went on to examine in which tissue the increased FGF21 serum levels were produced and found that loading induced a threefold increase in hepatic FGF21 expression, whereas the levels of FGF21 mRNA in WAT were not affected by loading, and the FGF21 mRNA levels in cortical bone and skeletal muscle were not detectable (Fig. 2C).

We also examined if an inflammatory response could be the reason for increased FGF21 levels in the load group by analyzing a range of cytokines in the serum of wild-type (WT) male mice, including IL-1*β*, IL-6, IL-10, IL-17A, interferon-*γ*, and TNF-*α*, but none of these markers differed between control and load mice (Fig. 2D).

### The BW-reducing effect of increased loading but not of leptin treatment is depleted in FGF21 transgenic mice with supraphysiological constant serum FGF21 levels

**Figure 3.**
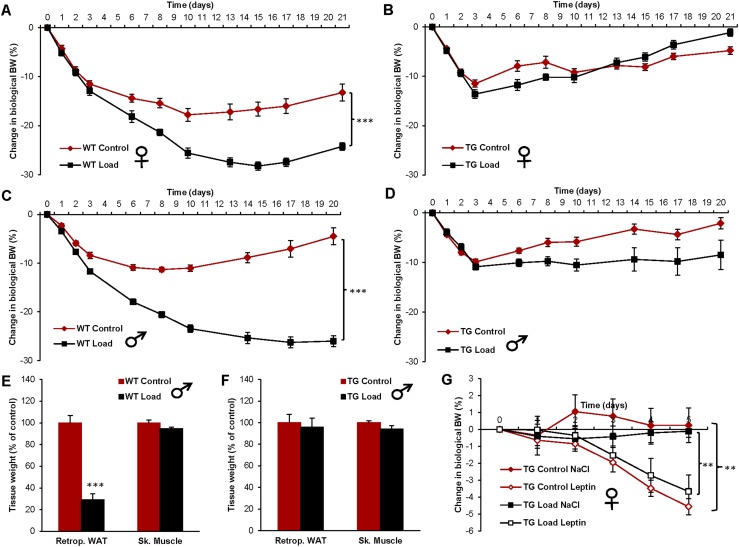
The BW-reducing effect of increased loading but not of leptin treatment is depleted in FGF21 transgenic mice with supraphysiological constant serum FGF21 levels. Effect of loading on BW change in (A) WT female mice (control n = 10, and load n = 10), (B) FGF21 transgenic (TG) female mice (control n = 9, and load n = 10), (C) WT male mice (control n = 10, and load n = 10), and (D) FGF21 TG male mice (control n = 9, and load n = 10). The effect of loading on tissue weights in (E) WT male mice and (F) TG male mice at day 21 after capsule implantation. (G) The effect of leptin treatment (1.5 µg/g BW twice daily) on changes in biological BW in female TG mice (TG Control NaCl n = 4, TG Control Leptin n = 5, TG Load NaCl n = 5, and TG Load Leptin n = 5), starting at day 21 after capsule implantation. Data are expressed as means ± SEM. **P < 0.01; ***P < 0.001. Retrop., retroperitoneal; Sk. Muscle, skeletal muscle (*musculus gastrocnemius*).

We next investigated if disruption of the normal regulation of FGF21 in FGF21-overexpressing mice (19) could interfere with the anti-obesity effect of loading. Compared with WT mice, the serum FGF21 levels were substantially increased in FGF21 transgenic mice (transgenic control 1285 ± 58 ng/mL and WT control 1.8 ± 0.3 ng/mL, *P* < 0.001). Furthermore, loading did not affect the serum FGF21 levels in the FGF21 transgenic mice (control 1285 ± 58 ng/mL and load 1248 ± 68 ng/mL, nonsignificant). Whereas loading markedly decreased BW in female WT mice (Fig. 3A), this effect was not seen in female mice with hepatic overexpression of FGF21 (Fig. 3B). The load-induced suppression of BW and body fat was also blocked in FGF21-overexpressing male mice (Fig. 3C–3E). Muscle mass was unaffected by loading in both WT and FGF21-overexpressing male mice (Fig. 3E and 3F). In contrast to increased loading, leptin treatment decreased BW in FGF21-overexpressing female mice, both in those without and those with simultaneous loading (Fig. 3G), demonstrating that the effect of increased loading but not of leptin treatment is blocked by supraphysiological FGF21 levels.

### The homeostatic regulation of BW and body fat by loading is functioning in FGF21 KO mice

**Figure 4.**
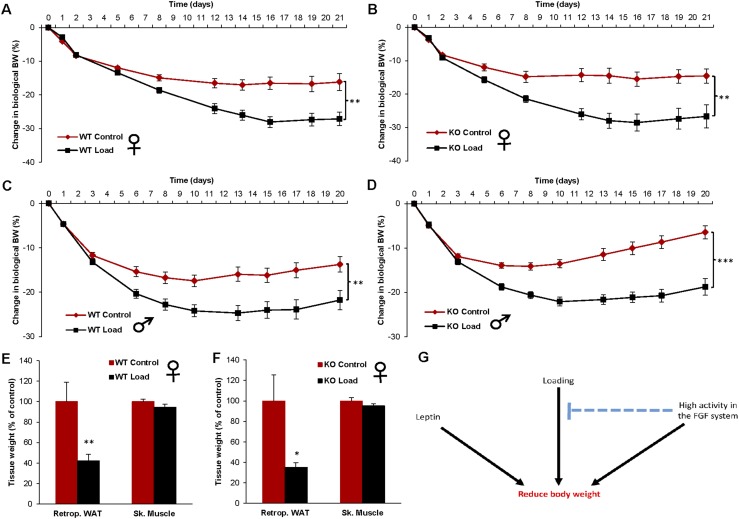
The homeostatic regulation of BW and body fat by loading is functioning in FGF21 KO mice. The effect of loading on BW changes in (A) WT female mice (control n = 10, and load n = 10), (B) FGF21 KO female mice (control n = 9, and load n = 10), (C) WT male mice (control n = 10, and load n = 10), and (D) FGF21 KO male mice (control n = 9, and load n = 10). The effect of loading on tissue weights in (E) WT female mice and (F) FGF21 KO female mice at day 21 after capsule implantation. Data are expressed as means ± SEM. *P < 0.05; **P < 0.01; ***P < 0.001. (G) Proposed interactions among leptin, increased loading, and the FGF system for the regulation of BW. Previous studies demonstrate that both loading and the FGF system have the capacity to regulate BW, independent of leptin (8–10). In the current study, we demonstrate that high activity in the FGF system, induced by overexpression of FGF21, blocks the BW-reducing effect of loading. In contrast, the effect of leptin on BW was not affected by altered activity in the FGF system, supported because overexpression of FGF21 did not affect the leptin response. However, FGF21 is not mediating the loading response, supported by a normal loading response in FGF21 KO mice. We propose that both the gravitostat and the FGF system regulate BW, independent of leptin, and that pharmacologically enhanced activity in the FGF system reduces the sensitivity of the gravitostat. Retrop., retroperitoneal; Sk. Muscle, skeletal muscle (*musculus gastrocnemius*).

To determine if FGF21 is mediating the effect of increased loading on BW, we added empty or loaded capsules to global FGF21 KO mice. The WT control females responded to loading with decreased BW (Fig. 4A), but also, the FGF21 KO females showed the same response to loading (Fig. 4B). We repeated the same loading experiment in FGF21 KO male mice and their WT littermates, resulting in a clear BW-suppressing response to loading in both WT (Fig. 4C) and FGF21 KO males (Fig. 4D). Furthermore, there was a substantial decrease in fat mass by loading in both WT and FGF21 KO female mice, whereas the muscle mass was unaffected by loading (Fig. 4E and 4F).

## Discussion

We have recently published data that reveal a BW homeostat, called the gravitostat, which senses the total weight and regulates BW and fat mass in rodents, with the expected accompanying effects on glucose metabolism (10, 11). The present results demonstrate that suppression of BW and body fat by increased loading is blocked in mice with pharmacologically stimulated FGFR1c signaling and in mice with overexpression of FGF21. However, the suppression of BW and body fat by loading was still present in FGF21 KO mice. Therefore, the loading effect on BW seems to be attenuated by high activity in the FGF system, although the presence of FGF21 does not seem to be essential for the loading effect.

Previous studies demonstrate that both loading and the FGF system have the capacity to regulate BW, independently of leptin (8–10). We herein investigated the effect on loading in interaction with the FGF system. It has been documented by our group and others that ligands modulating the FGFR1c activity exert a marked body fat-reducing effect in mice (8, 9), and there is also evidence that this system is important for regulation of BW in humans (20, 21). In the current study, we investigated if an FGFR1c antibody with a known pronounced anti-obesity effect (8) could interfere with the BW-suppressing effect of increased loading. IgG-treated control mice responded to loading with a decreased BW and body fat, whereas treatment with an antibody directed against FGFR1c completely blocked these differences in BW and body fat, supporting the notion that the FGF system has the capacity to interact with the loading response.

Based on the findings that interference with FGFR1c blocked the homeostatic regulation of BW by increased loading and that circulating liver-derived FGF21 decreases BW via central FGFR1c-*β*-Klotho signaling (21–25), we next evaluated the effect of increased loading on three endocrine FGFs: FGF15, FGF21, and FGF23, which have previously been shown to interact with metabolism (23, 26, 27). Whereas FGF23 levels were not affected (10), FGF15 levels were moderately decreased by loading. Like FGF21, FGF15/19 seems to regulate metabolism and body composition, but the effects might be exerted more on lean body mass rather than on fat mass (27). Importantly, we found evidence that loading increased serum FGF21 levels and hepatic FGF21 expression. The mechanism behind this effect is largely unknown. It has been reported that inflammation increases FGF21 production (28). However, inflammation is unlikely to cause the loading-induced FGF21 production seen here, given that the levels of several inflammation-related cytokines were unchanged by loading in the current study (Fig. 2D).

Given that pharmacological FGF21 treatment has the capacity to reduce fat mass (21, 29), we next investigated if disruption of the normal regulation of FGF21 in FGF21-overexpressing mice could interfere with the anti-obesity effect of loading. To this end, we used FGF21 transgenic mice with substantially augmented and loading-independent FGF21 expression under the nonphysiological control of the apolipoprotein E promoter (19). The load-induced suppression of BW, seen in WT mice, was blocked in FGF21-overexpressing mice of both genders. The BW of FGF21 transgenic mice was lower than that of WT mice (Table 1). This is unlikely to be the reason for the lack of suppression of BW by loading, given that body growth and bone length, rather than fat mass, are decreased in FGF21 transgenic mice (19). Moreover, to evaluate if the lack of BW suppression was a result of a general resistance to weight loss, we treated the FGF21 transgenic mice with leptin. In contrast to loading, leptin treatment decreased BW in FGF21-overexpressing mice independent of loading, demonstrating that they did not have a general resistance to weight loss.

The serum levels of FGF21 in the FGF21 transgenic mice were at similar levels as previously reported for these mice (30) and substantially higher than in both WT control and WT load mice. These levels could be regarded as a high pharmacological dose of FGF21, and we propose that these pharmacological levels could lead to a desensitization of the gravitostat. Furthermore, it may not be discarded that at such a high concentration, other receptors of the FGF family may be activated as well. Potential side-effects of these high FGF21 concentrations could be decreased bone mass (31) and infertility in female mice (32). However, the response to high chronic doses of FGF21 was aborted in FGFR1 KO mice (33), indicating that the FGFR1 probably is the main receptor mediating the effects of FGF21.

To determine if altered FGF21 expression mediates the loading response, we added empty or loaded capsules to global FGF21 KO mice. FGF21 KO mice of both genders displayed a normal loading response, demonstrating that altered FGF21 expression is not mediating the loading response and that FGF21 is not essential for a functional loading response.

In the current study, we found evidence that FGF21 gain-of-function models attenuate the BW loss in response to loading. Loading and FGF21 seem to influence body fat mass via different mechanisms. We have previously shown that the loading of mice mainly decreases body fat by decreasing food intake but does not alter energy expenditure (10), whereas FGF21 seems to increase mainly energy expenditure rather than food intake (24). Therefore, FGF21 and loading seem to influence body fat mass by different mechanisms, and the reason for the interference between FGF21 and loading is still unclear.

In conclusion, previous studies demonstrate that both loading and the FGF system have the capacity to regulate BW, independent of leptin (Fig. 4G) (8–10). In the current study, we demonstrate that high activity in the FGF system, induced by overexpression of FGF21, blocks the BW-reducing effect of loading (Fig. 4G). In contrast, the effect of leptin on BW was not affected by altered activity in the FGF system, supported because overexpression of FGF21 did not affect the leptin response (Fig. 4G). However, FGF21 is not mediating the loading response, supported by the normal loading response in FGF21 KO mice. We propose that both the gravitostat and the FGF system regulate BW independent of leptin and that pharmacologically enhanced activity in the FGF system reduces the sensitivity of the gravitostat (Fig. 4G).

## Abbreviations:

BW: body weight
FGF: fibroblast growth factor
FGFR: fibroblast growth factor receptor
KO: knockout
PGK-Cre: mice expressing Cre recombinase under the control of the phosphoglycerate kinase-1 promoter
WAT: white adipose tissue
WT: wild-type
